# Common ground in Attention Deficit Hyperactivity Disorder (ADHD) and Borderline Personality Disorder (BPD)–review of recent findings

**DOI:** 10.1186/2051-6673-1-3

**Published:** 2014-04-10

**Authors:** Swantje D Matthies, Alexandra Philipsen

**Affiliations:** Department of Psychiatry & Psychotherapy, Clinic of Mental illnesses, University Medical Centre, Hauptstr. 5, D-79104 Freiburg, Germany

**Keywords:** ADHD, BPD, Impulsivity, Emotion regulation

## Abstract

Considerable overlap in diagnostic criteria and shared psychopathologic symptoms in attention deficit hyperactivity disorder (ADHD) and borderline personality disorder (BPD) have stimulated research activities in this field. Longitudinal studies have shown that BPD is frequently diagnosed in adult patients who had been diagnosed with ADHD in childhood. The question of whether ADHD and BPD randomly co-occur as comorbidities, have similar origins or share common pathological mechanisms remains unresolved. Some authors suggest that ADHD contributes to the development of BPD via various mechanisms, and therefore consider it a risk factor for later BPD development.

In this article the evidence for the co-occurrence of these disorders will be reviewed as well as studies on their common genetic and environmental influences. Temperamental and developmental issues will be reviewed, and shared features such as impulsivity and emotion dysregulation discussed. From a therapeutic perspective, few studies have investigated psychotherapeutic treatment of the comorbid condition, though the issue is highly important to the management of patients suffering from both disorders. Some thought is given to how therapeutic methods and approaches can be modified to benefit patients, and to their possible succession.

## Introduction

In recent years, shared symptoms and considerable overlap in clinical presentation between attention deficit hyperactivity disorder (ADHD) and borderline personality disorder (BPD) have stimulated research activities in this field. Since the 1980s numerous studies have furnished evidence on the persistence of ADHD symptoms in adulthood [1–6]. ADHD symptoms consequently have been increasingly recognised in BPD patients. It has become common knowledge that both disorders frequently occur as life-long comorbidities [7] and often result in serious health related consequences with regards to psychopathology, quality of life and psychosocial functioning.

On the one hand, since the publication of the Wender Utah Criteria [8] emotion dysregulation - the core symptom of BPD–has been widely recognised as an important symptom in adult ADHD. The current discussion on emotion dysregulation as an important feature of ADHD in adults [9–11] further spotlights the common ground between the disorders. On the other hand, as well as the evident absence of inattentional symptoms in BPD–with the exception of dissociative states–differences between ADHD and BPD in neuropsychological organisation and functioning have increasingly become evident [12, 13].

Discussion is continuing on whether ADHD and BPD occasionally co-occur as comorbidities and have common origins, possibly with ADHD traits being the precursor of later BPD, or share common pathological mechanisms. Against this background there has been discussion about which therapeutic approach would be the most beneficial, even if research activities in this field are still scarce.

This article gives an overview of current knowledge about the close links, subtle distinctions and significant differences between the two disorders. It deals with different aspects of the association between ADHD and BPD, taking into account possible common origins in neurobiology and inborn temperament as well as common mechanisms concerning neuropsychology and emotion regulation. As we consider this a very important issue to improve and broaden knowledge of therapeutic options for patients with both ADHD and BPD, this basic information is provided as a starting point for clinicians dealing with patients suffering from both conditions. The article will also give some thought to therapeutic aspects and formulate some tentative recommendations.

### Diagnostic criteria: overlap in clinical symptom presentation

BPD is classified as a personality disorder (PD). The DSM-V defines the main features of BPD as a “pattern of instability in interpersonal relationships, self-image, and affects, and marked impulsivity” [14]. ADHD according to the DSM-V is a neurodevelopmental disorder characterized by “a persistent pattern of inattention and/or hyperactivity-impulsivity that interferes with functioning or development” [14]. Thus, at first glance the overlap in diagnostic criteria is primarily in the domain of impulsivity. Pursuant the ICD-classification [15], impulsive behaviors are characteristic for both subtypes of the so-called emotionally instable personality disorder (see the list of characteristics of the impulsive subtype of emotionally instable personality disorder according to the ICD):

- Marked tendency to act unexpectedly and without consideration of the consequences,
- To engage in quarrelsome behavior and to have conflicts with others, especially when impulsive acts are thwarted or criticized,
- Liability to outbursts of anger or violence, with inability to control the resulting behavioral explosions,
- Difficulty in maintaining any course of action that offers no immediate reward and
- Unstable and capricious (impulsive, whimsical) mood

The criteria of the impulsivity domain in ADHD according to the DSM-V are quite similar (see the criteria of the impulsivity domain of ADHD according to DSM-V):

- Difficulty waiting his or her term (e. g. while waiting in a line)
- Blurting out answers before a question has been completed or blurting out inappropriate comments without regard to consequences and
- Interrupting or intruding on others

Because of this apparent overlap the phenomenological differentiation between “borderline-impulsivity” and “ADHD-impulsivity” is merely possible from a clinical viewpoint. Both patient groups often seem to act without taking into consideration the consequences of their behavior. According to the ICD definition of the impulsive subtype of emotionally instable PD, the cited impulsivity criteria also comprise emotion control/regulation difficulties (outbursts of anger). The aspect of emotion dysregulation was added to the definition of adult ADHD when the Wender-Utah criteria were published. Wender et al. proposed a set of characteristics (see the list of additional Wender Utah criteria of adult ADHD):

- Affective lability or mood shifts
- Hot temper outbursts or short-lived explosive outbursts or constant irritability and
- Emotional over reactivity in the sense of over reactivity to routine life stress

to specify current ADHD symptoms in adults which are not only based on the behavioral criteria outlined in DSM-V, but also comprise associated features and subjective symptoms [16].

Thus, it is evident that there is a descriptive overlap with the affective instability domain in BPD. Wender’s proposal has been tested, and evidence has emerged to affirm that in adult ADHD there is an elevated risk for deficient emotional self-regulation (DESR) [9, 17–19]. A recent family study reports the finding that ADHD with DESR might either represent a distinct familial subtype of ADHD or an entirely separate familial condition [11].

The considerable overlap between symptoms of ADHD and BPD in the core domains of impulsivity and emotional dysregulation led to the idea that both disorders might be the result of different developmental pathways based on common underlying pathologic mechanisms and differing in symptom severity [20].

Psychopathological presentation was therefore investigated in adult patients with ADHD compared to BPD patients and control subjects with a focus on Borderline symptomatology [21]. Severity of borderline symptoms was assessed with the borderline symptom list (BSL). Adult ADHD patients showed significantly higher BSL total scores compared to healthy controls but lower scores than BPD patients. The largest differences between ADHD and BPD patients were found with respect to self-destruction and affect dysregulation, whereas the smallest difference was found with respect to loneliness. Thus, at a descriptive level symptoms of BPD are common to ADHD patients, but are less severe.

**Figure 1:**
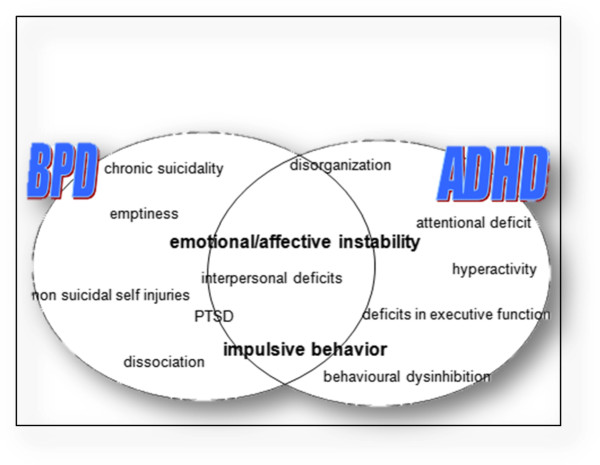
**Shared features in ADHD and BPD.**

Given the similarities in symptom presentation, one might think that ADHD and BPD are different modes of the same disorder. However, states of inner tension [22] which are often regulated through self-injurious behavior and repeated suicidal ideation, as well as temporary stress-related paranoid ideation or dissociative symptoms, are typical of BPD but not of ADHD. Inadequate efforts to prevent abandonment in intense but instable relationships, marked instability of identity and extreme alterations in thinking (devaluation vs. idealization) as well as feelings of emptiness are features of BPD [14]. These severe symptoms rarely occur in ADHD patients whereas inattention and hyperactivity, the core symptoms of ADHD, are not considered typical of BPD patients suggesting marked differences between the disorders Figure 1.

## Method

We searched relevant registers for studies on ADHD. Furthermore, we screened the reference lists of relevant articles manually. Included were papers in English, German and French. Studies of any design focusing on ADHD and BPD were screened and categorized. We used the categories research on comorbidity, etiopathology, temperament and development, neuropsychology and treatment to summarize the findings.

A search for studies dealing with ADHD and BPD was conducted for the following bibliographic databases: Pubmed, Embase, Medline, PsychInfo, Central (The Cochrane Central Register of Controlled Trials). The following terms were used: (ADHD OR (attention deficit) OR (attention-deficit) OR hyperactivity*) AND (BPD OR (borderline personality disorder) OR borderline*). The search included all fields in Pubmed, Embase, Medline and PsychInfo databases and abstract, title and keywords in the Central bibliography (number of citations: Pubmed 444, Embase (ScienceDirekt): 11660, Medline (ME95) see Pubmed, Psychinfo 418, Central (The Cochrane Central Register of Controlled Trials) 19, NCBI webside 420).

## Review

### ADHD and BPD as comorbidities

By 1979, a connection between minimal brain dysfunction and BPD was assumed to exist [23]. Investigating the natural course of childhood psychiatric disorders in a sample of young adults, a relationship between disruptive disorders and cluster B personality disorders, namely between childhood ADHD and later BPD, was reported in 1995 [24]. PDs have been found in follow-up studies of patients with childhood ADHD [25, 26]. Fischer and colleagues reported that 14% of a young adult follow up sample of hyperactive children had developed BPD vs. 3% in the control group [25]. In another longitudinal study adolescents with ADHD were significantly more likely than controls to be diagnosed with BPD (13.5% vs. 1.2% in the control group, odds ratio OR = 13.16). Those who continued to meet diagnostic criteria for ADHD at follow up had a higher rate of BPD diagnosis than those who remitted and controls (19% in “persisters” vs. 6.3% in “remitters”, [27]).

In adult ADHD, PDs are frequent comorbidities [28, 29]. Jacob et al. found that 27.2% of 372 adult ADHD patients suffered from a BPD if examined with the Structured Clinical Interview for DSM-IV (SCID II) [28]. As evaluated with the International Personality Disorders Examination (IPDE), 18.3% of an adult ADHD sample had a BPD diagnosis and severity of childhood ADHD symptomatology was associated with a higher frequency of PD diagnoses in adulthood [30]. Cumyn and colleagues report on a sample of 447 adults comparing subtypes of ADHD with regard to the comorbidity pattern. In this sample women with ADHD were more likely to have BPD than men. BPD was found in 24% of the combined type ADHD patients and in 10% of the inattentive ADHD patients [31]. Particularly in forensic populations, BPD diagnoses are frequent in subjects suffering from ADHD [32–34]. Female offenders with ADHD suffered more often from BPD (63.6%) than offenders without ADHD (25.3%) [33]. Fossati et al. investigated the association between childhood ADHD and BPD in adulthood. They reported 59.5% of the adult BPD patients scored above the cut-off score on the Wender Utah Rating Scale (WURS) measuring childhood ADHD symptoms retrospectively [35]. The percentage was considerably higher than in patients with other Cluster B personality disorders (10.6%), in patients with cluster A and C personality disorders (10.5%), in patients not suffering from a PD (5.8%) and in non-clinical subjects (6.5%). Prevalence of persisting combined-type ADHD in women with BPD was 16.1% whereas 41.5% had had ADHD in childhood [36], suggesting that childhood ADHD is a risk factor for the development of BPD in adolescence and adulthood. Since that study, the association between ADHD and BPD has been confirmed in a large national sample of the US population in which 34,000 adults aged an average of 18.4 years participated in face-to face surveys (National Epidemiologic Survey on Alcohol and Related Conditions (NESARC), [37]). Lifetime comorbidity with BPD was 33.69% in the ADHD individuals compared with 5.17% in the general population, resulting in an OR of 2.84 if adjusted for sociodemographic characteristics and other psychiatric disorders.

### Etiopathology

The association between ADHD and BPD has been primarily studied on a phenotypic level. Recently a study investigated the genetic and environmental contributions to this association using a dimensional approach. Borderline personality traits and ADHD symptoms were assessed in a sample of 7233 twins and siblings from the Netherlands. The phenotypic correlation between ADHD and BPD symptoms was high (r = 0.59) and was the same for both genders. According to the authors, 49% of the high phenotypic correlation can be explained by genetic influences and 51% by environmental factors [38]. It seems conceivable that common biological factors influencing both ADHD and BPD symptoms play a role in these overlapping psychopathological domains.

#### Environmental influences

To study environmental influences on ADHD symptoms, Lehn and colleagues conducted a longitudinal study in monozygotic twins [39]. Twins with more pronounced ADHD traits had lower birth weight and delayed physical growth and motor development than unaffected twins. Maternal smoking, sleeping in different rooms, and living with only one parent were environmental factors of influence concerning concordant ADHD traits [39]. For BPD, a retrospective assessment of prenatal adversities revealed that BPD patients were more often exposed to adverse intrauterine conditions than a matched control group. Prenatal tobacco exposure (OR = 3.37) and medical complications (OR = 2.87) were important risk factors for later BPD. Separate analyses revealed that these findings were not a result of comorbid ADHD diagnoses [40].

#### Traumatic experiences

Other environmental risk factors that contribute to or influence the development of BPD include traumatic experiences. It has been found that the lifetime prevalence of Posttraumatic Stress Disorder (PTSD) and BPD in a nationally representative sample of the U. S. population is 6.6% and 5.9% respectively (National Epidemiologic Survey on Alcohol and Related Conditions (NESARC) Wave II, N = 34.653, [41]). PTSD and BPD had a high degree of lifetime co-occurrence: of individuals with BPD, 30.2% were also diagnosed with PTSD, whereas 24.2% of individuals with PTSD were also diagnosed with BPD. Individuals with comorbid PTSD-BPD had a higher prevalence of repeated childhood traumatic events than individuals with either condition alone [41]. A review of the literature concerning the association between traumatic experiences and development of BPD concludes that the data available to date are supportive of childhood trauma as an etiologic factor in BPD [42].

A recently published large longitudinal study followed children with and without ADHD and their siblings for 10 years (mean age at follow-up: 22 years). This study revealed that ADHD was an independent risk factor for later PTSD with an OR of 2.23 [43]. Rucklidge et al. administered the Childhood Trauma Questionnaire (CTQ) to an adult ADHD sample, allowing for the retrospective assessment of traumatic experiences in childhood, and found significantly higher CTQ total scores in the ADHD group than in a control group. In particular, emotional abuse and neglect were frequent in ADHD patients, and sexual abuse and physical neglect were more frequently reported by women with ADHD [44]. Philipsen and colleagues confirmed a strong association between the retrospective diagnosis of childhood ADHD in BPD women and reported emotional abuse in childhood as measured by the CTQ [36]. Nonetheless, the interrelation between ADHD symptomatology, traumatic experiences and BPD remain the subject of debate. Difficult parent-child relationships caused by ADHD symptoms present in the child and possibly also in the parents might predispose to traumatic interactions and favor emotional abuse and neglect [45]. Risky, impulsive and novelty seeking behaviors in ADHD children might heighten the risk for exposure to traumatic situations. Conversely, it could be argued that experiencing a trauma may lead to more severe ADHD symptoms. We speculate that ADHD children are at elevated risk for adversities and traumatic experiences in childhood and that this contributes to the development of BPD in adolescence and adulthood. It seems plausible that the interaction between a “sensitive” genotype or risk factor (i.e., the genetic predisposition to ADHD) and non-fitting environmental influences in childhood may lead to the development of BPD in adulthood, especially when parents are themselves impaired by symptoms of ADHD [38].

### Temperament and development

Predictive data concerning the development of BPD comes from prospective studies. Burke and Stepp, for example, report on longitudinal data from a clinical sample of 177 boys, first diagnosed at ages 7 to 12 and followed up annually to age 18 and reassessed at age 24 [46]. Diagnoses of ADHD and oppositional Defiant Disorder (ODD) in childhood were predictive of later BPD symptoms [46, 47]. In a recent review, Martel [17] proposes several models elucidating the development of associations between emotion-regulation related temperament/personality traits and disruptive behaviours. The proposed models delineate possible developmental pathways, and serve as starting points for the explanation of comorbid disorder development. Martel concludes that “high negative and positive emotionality may be associated with disruptive behavior disorders in general, deficient control processes may be specifically associated with ADHD, and low agreeableness may be specifically associated with oppositional-defiant and conduct problems”. More specific linkages between emotion-regulation characteristics and psychopathologic domains can be seen between low effortful control and inattention, and between high emotionality and hyperactive-impulsive symptoms. The emotion-related traits seem to be related to oppositional defiance and conduct problems in a developmental progression [17].

A study of 103 female adult patients meeting DSM-IV criteria for ADHD or BPD used a latent class analysis (LCA) and identified four subgroups. Hyperactivity was present in all four subgroups and thus does not discriminate between ADHD and BPD. Developmental pathways were also investigated. One possible pathway leads from combined ADHD symptoms or mainly inattention symptoms in childhood to an adult profile containing only symptoms of ADHD and not BPD. Another possible pathway leads from a childhood profile with low levels of hyperactive/impulsive symptoms but no inattention problems to an adult profile with symptoms of BPD [48]. Temperament and character traits are hypothesized to play an important role in the developmental pathways leading to ADHD and/or BPD. In the same study, Cloninger’s temperament and character traits were also assessed. Cloninger defines dimensions of temperament (harm avoidance, novelty seeking and reward dependence) and dimensions of character (self-directedness, cooperativeness and self-transcendence). The LCA revealed a high novelty seeking temperament in all detected latent classes except for the class with a preponderance of BPD symptoms. Patients with both BPD and ADHD symptoms (all symptom domains) scored highest for novelty seeking. Higher than normal scores in harm avoidance were found in all patients with ADHD and BPD symptoms, whereas patients with ADHD symptoms only did not have elevated harm avoidance scores. Self-directedness and cooperativeness were lower than normal in patients with BPD symptoms. Van Dijke et al. concluded that above-average novelty seeking temperament is more strongly linked to ADHD than to BPD. Harm avoidance, in contrast, seems more likely to be linked to BPD. Thus, ADHD patients score high on Cloninger’s temperament scales whereas BPD patients also show abnormal scores on the character scales low self-directedness and cooperativeness, a finding which points to the difference in conceptualization between the disorders: the concept of ADHD should therefore not be misclassified as a personality disorder [49].

### Neuropsychology

#### Impulse control

Of the official diagnostic criteria defining ADHD and BPD, the highest overlap between the two disorders seems to be in the domain of impulsivity. But what is meant by impulsivity? A comprehensive definition of impulsivity does not exist. The presentation of impulsive behaviour is manifold, and comprises behaviours that often result in undesirable outcomes because they are not sufficiently thought-out or inappropriate or occur in an untimely fashion [50–53]. Impulsive behaviour refers not only to actions that are mistimed but also to actions that are difficult to control. A core component of impulsivity in ADHD from a neuropsychological viewpoint seems to be difficulty in inhibiting responses [54], as demonstrated by slower reactions to stop signals in stop-signal tasks [55]. Nigg et al. showed that symptoms of BPD also correlated with response inhibition, even after controlling for ADHD [56]. A deficit of response inhibition in BPD was also reported in an auditory version of a Go/Nogo paradigm [57]. Recent studies have found mixed results, some confirming inhibition deficits in BPD [58, 59], but others not finding significant differences in comparison to depressive patients and to a healthy control group [12, 60, 61]. Jacob and colleagues reported significantly higher scores on self-report measures of impulsivity in BPD, but not in behavioural tests. Behavioural inhibition errors were correlated with more intense emotional states [62]. Lampe et al. [12] compared inhibitory functions in patients with ADHD, patients with BPD, patients suffering from both disorders and healthy controls. Patients with ADHD, either alone or paired with BPD, scored higher on the Barratt Impulsiveness Scale than pure BPD patients and controls (which did not differ). ADHD patients performed worse than BPD patients in two inhibitory tasks, whereas the comorbid group showed deficits in only one inhibitory task. The finding of higher scores on psychometric impulsivity measures in a BPD-ADHD-group compared to a BPD only group was confirmed by Ferrer et al. [63] and Speranza and colleagues [64]. In a recent review Sebastian et al. [13] present an overview of the role of ADHD concerning impulsivity in BPD, and come to the conclusion that high impulsivity might be especially present in the comorbid group of patients suffering from both ADHD and BPD.

Moreover, Krause-Utz and colleagues reported stress-dependent increase in state impulsivity in patients with BPD (both alone and comorbid with ADHD) in comparison to an ADHD only group and healthy controls [65]. BPD seems to involve impulse control problems when affective or interpersonally sensitive aspects are involved, whereas emotionally neutral impulse control seems less affected [13]. In ADHD, on the other hand, a more generalized attentional or cognitive processing deficit seems to account for problems in behavioral inhibition [55]. Motivational aspects [66] and high emotionality [17] add to the high amount of impulsive behavior due to executive function alterations in ADHD.

#### Emotion regulation

As emotion dysregulation is often considered the core feature of borderline psychopathology, components of emotion dysregulation have been intensively studied in BPD (for Review see [67]). Evidence suggests that individuals with BPD frequently show maladaptive emotion regulation strategies as rumination [68] and suppression [69], and also impulsive behaviors and suicidal and self-injurious behaviour [70–72]. Linehan’s biosocial theory [73] suggests that states of unbearable tension [22] develop as a cascade out of highly intensive negative affects developing suddenly [74], and low levels of distress tolerance [75]. Greater polarity of affect has been reported in BPD patients [76]. In addition a high emotional sensitivity has been assumed as a dispositional feature of BPD patients. In a longitudinal study from child–to adulthood, sociability and impulsivity in early childhood–as a factor possibly influenced by emotional sensitivity–was correlated to borderline symptomatology in adulthood [77]. Some studies have found slower reaction times in the emotional Stroop Task, suggesting higher emotional sensitivity in BPD [78, 79]. Beyond that a negativity bias in emotion recognition has been hypothesized in BPD individuals (for Review see [80]). Taken together, emotion regulation in BPD seems to be impaired in a very complex interplay between high emotion sensitivity, high propensity to negative affect and a deficit in the ability to appropriately regulate negative states due to low distress tolerance and a tendency toward maladaptive emotion regulating behaviors [67].

In ADHD research evidence in the field of emotional dysregulation is relatively sparse. Negative emotions seem to contribute to or aggravate ADHD symptoms [17, 81] via assumed emotion regulation deficits [17, 81–85]. Sjöwall et al. [86] provided evidence for the central importance of emotion regulation difficulties in ADHD children independent of neuropsychological impairments. However, the nature of deficient emotional self-regulation in ADHD is unknown [87]. It might be characterized by deficits in regulating the physiological reactions following emotions, problems inhibiting uncoordinated behavioral tendencies and expressions caused by emotions, and/or difficulties controlling and refocusing attention when emotional reactions have been triggered [88]. Bresner et al. investigated emotion-regulation difficulties (measured psychometrically) in adult patients suffering from ADHD in comparison to age matched control subjects and found the following characteristics: ADHD patients differed significantly from control subjects concerning the intensive feeling to be overwhelmed by emotions and had less capacity to regulate and control emotions. Moreover ADHD patients reported a significantly higher tendency toward rumination and resignation [83]. Therefore not only the perception of acute emotional states but also the regeneration process from emotional states seems to be impaired via rumination pathways.

It can be assumed that individuals suffering from ADHD, easily overwhelmed by emotions, might struggle with emotional regulation strategies designed to suppress the expression of emotion. Evidence comes from a study of boys suffering from ADHD compared to a control group. Both groups were observed during a frustrating competitive Puzzle Task and randomised to direct instruction to either control their emotional expressions or let them run their natural course. The ADHD group was not as successful in doing so as the control sample. In the ADHD group the difference in emotional dysregulation between the condition instructed to control their emotions and the natural course condition was not as pronounced as in the control group. The effect of suppression on the level of emotional dysregulation in ADHD children was rather small, which points to the conclusion that ADHD children are less effective in suppressing their emotional expressions [85].

It is currently not possible to compare emotion regulation in BPD and adult ADHD because more evidence for its role in ADHD is required, and no direct comparison studies have been carried out Table 1.

**Table 1:** **Overview on reviews and studies concerning emotion regulation in ADHD and BPD**

Author	Design	Population	Description	Result
Berger et al. [82]	Review article	ADHD (developmental perspective)	Reviews empirical findings concerning selfregulation by focusing on	-
			ADHD, including a detailed review of animal models related to the disorder.	
Bresner et al. [83] (Dissertation, in German)	Questionnaire study	Adults with ADHD (*n* = 50) and control subjects (*n* = 67)	Self-ratings concerning the experience and control of emotions, state and trait anger, coping behavior and also neuropsychological assessment of attention.	ADHD patients reported more emotion regulation and coping problems. They reported a tendency to rumination and resignation as well as the feeling of being frequently overwhelmed by their emotions.
Carpenter & Trull [67]	Review article	BPD	Reviews scientific evidence for the components of Linehan’s biosocial theory (emotion sensitivity, heightened and labile negative affect, deficit of appropriate regulation strategies, surplus of maladaptive regulation strategies).	-
Desman et al. [84] (in German)	Neuropsychological laboratory study with go-/nogo-task modified from the Testbattery for Attentional Performance	Boys with ADHD (*n* = 19) and control boys (*n* = 19)	Five conditions of the task: neutral, feedback, reward only, response cost only, reward, and response cost. Performance, emotional well-being and coping were recorded.	Boys with ADHD showed an age-dependent deficit in behavioral inhibition as well as reduced attention independent of condition. Boys with ADHD reported increased challenge and more rumination.
Martel [19]	Review article	ADHD (developmental perspective)	Literature review on studies addressing relations among emotional regulation, disruptive behavior disorders, and ADHD.	-
Marx et al. [81]	Laboratory study, emotional working memory task (n-back task)	adults with ADHD (*n* = 39), matched control subjects (*n* = 40)	In the background of the working-memory task stimuli that varied in emotional saliency which had to be ignored during the task.	ADHD patients showed a general working memory deficit and enhanced distractability by emotionally salient stimuli.
Musser et al. [87]	Laboratory study, assessment of physiological data	Children with ADHD (*n* = 32) and control children (*n* = 34)	Parasympathetic and sympathetic nervous system reactivity was measured (respiratory sinus arrhythmia–RSA, cardiac pre-ejection period–PEP) during an emotion task with four conditions (2 valences and 2 emotion regulation strategies): induction of negative affect, suppression of negative affect, induction of positive affect, suppression of positive affect.	Group differences parasympathetic regulation (RSA), no group differences in sympathetic activity (PEP). Control children showed adaptive alteration of RSA depending on emotional valence and emotion regulation strategy. Children with ADHD showed elevated parasympathetic activity across all task conditions compared to baseline.
Rash & Aguirre-Camacho [88]	Systematic review	Children with ADHD	Review of studies examining the relationship between cardiac vagal control and ADHD.	-
Sjöwall et al. [86]	Laboratory study with neuropsychological assessment and parent ratings	Children with ADHD (*n* = 102) and a matched control sample.	Executive functions (working memory, inhibition, and shifting), delay aversion, and reaction time variability were measured. Parent ratings of emotion regulation and a test of emotion recognition (facial expressions) were included.	Children with ADHD differed significantly from controls on all measures, except for delay aversion and recognition of disgust.
				Executive functioning, reaction time variability, and emotional functioning (especially anger regulation, anger recognition, and regulation of happiness) contributed independently to distinguishing between children with ADHD and controls.
Walcott & Landau [85]	Laboratory study, neuropsychological and behavioral data assessment	Boys with ADHD (*n* = 26) and control boys (*n* = 23)	A frustrating competitive puzzle task was administered as emotion regulation task. Half of all boys in each group were explicitly instructed to hide their feelings during the competition. Behavioral inhibition was examined using the Stop Signal Task	ADHD boys showed more disinhibition and less effective emotion regulation than comparison boys. They were unsuccessful in hiding their emotions.
			(SST), and emotion regulation was assessed via structured observation data.	Comparison boys were more successful in self-regulation. There was a relationship between emotion regulation competencies and effectiveness of inhibition.

### Treatment

In clinical practice, ADHD may frequently be missed in patients with a diagnosis of BPD. As ADHD is a frequent comorbidity in BPD and entails additional problems and impairing consequences in daily life, in therapeutic contexts careful diagnostic assessment of symptoms and problems is necessary. In each individual patient a decision about the necessity of treating the ADHD symptoms needs to be made based on a balanced evaluation of the severity of impairments due to ADHD, the patient’s resources, compensatory strategies and the patient’s demands [89]. Frequently in clinical practice, inattention and hyperactivity lead to difficulties in the acquisition of knowledge and skills necessary to deal with the impairing consequences of both disorders. Therefore treatment of comorbid ADHD in BPD patients possibly enhances psychotherapeutic outcomes.

#### Pharmacological strategies

ADHD in adults can be treated effectively with stimulant medication and atomoxetine. International guidelines propose methylphenidate (MPH) as the first line treatment in adult ADHD [89–91]. MPH treatment of adult ADHD should be part of a comprehensive treatment program that also addresses psychological and social aspects [92].

Case-reports of successful methylphenidate treatment in patients with co-morbid BPD and ADHD have been published [93, 94]. Golubchik and colleagues assessed the efficacy and tolerability of MPH treatment in fourteen adolescent females with ADHD and BPD in an open study. Participants received MPH for 12 weeks. Significant improvement was detected not only for ADHD but also for BPD severity and aggressive behavior. A positive effect on self-injurious behavior was observed [95]. Despite the observed positive effects of MPH treatment in patients suffering from BPD and ADHD, careful evaluation and monitoring is necessary because of the high number of concomitant substance abusers. If methylphenidate is not well tolerated or if there is concern about the potential for drug misuse and diversion, atomoxetine or noradrenergic or dopaminergic antidepressants are alternatives [96–98]. As well as pharmacological treatment, psychotherapeutic options should be discussed.

#### Psychotherapeutic options

It can be assumed that BPD patients suffering from ADHD are hindered in the accomplishment of psychotherapeutic steps because ADHD implicates difficulties in planning, structuring, concentrating, arranging timetables and pursuing schemes. Motivation is often fragile, initial enthusiasm is followed by frustration and boredom, and focusing on central themes often remains a challenge. To avoid early discontinuation of treatment and disappointment, ADHD should be kept in mind and included in the planning of treatment steps.

BPD traits [99] and BPD are mainly treated with psychotherapy. Four efficient disorder-specific psychotherapeutic approaches are known; cognitive-behaviourally oriented dialectical behavioural therapy (DBT), schema-focused therapy (SFT), the psychodynamically oriented transference focused psychotherapy (TFP) and mentalisation-based treatment (MBT). DBT has been studied intensely and beneficial effects in BPD for anger reduction, parasuicidality and mental health have been shown [100–104], for Cochrane Review see [105].

For adults with ADHD a number of different psychotherapeutic programs are available, mostly group therapies (for Review see [106–108]). An adaptation of DBT skills training for adults suffering from ADHD has been developed [109–112]. The group therapy program [113] has been developed for outpatients but can be easily adapted for individualized therapy sessions. Modules include mindfulness training to become aware of attentional and cognitive processes, and to learn to focus attention on the relevant task. Distress tolerance skills are taught to reduce hyperactivity, disorganized behavior, impulsivity and affective impairments. Emotion regulation skills help to control emotions and overexcitability. To address the frequent relationship problems suffered by individuals with ADHD, interpersonal effectiveness skills are taught. The emotion regulation modules have been evaluated as very helpful by group program participants [111]. From a clinical perspective, patients with co-morbid ADHD and BPD suffer from some additional problems, mainly in the domains of inattention, understimulation and novelty and risk seeking behaviour (amongst others). Psychotherapists should understand these co-morbid conditions, and consider the implications of the interaction between BPD and ADHD, for example to identify ADHD-typic antecedents of dysfunctional behavior (e.g. under-stimulation) or ADHD-specific handling of displeasing situations (e. g. hot temper).

A systematic approach to treatment can be proposed, including:

- Psychoeducation on both disorders and treatment options;
- Pharmacological treatment of ADHD symptoms if their severity indicates a treatment and if psychotherapeutic approaches might be facilitated by treating ADHD conditioned impairments. Stimulants are the treatment of choice for adults with ADHD, with long-lasting, extended release formulations preferred to avoid rebound with the risk of enhancement of mood swings and dysfunctional behavior, and for protection against abuse [91];
- Coaching on ADHD related therapy-interfering behaviors (e. g. shortage of patience and perseverance, tardiness, learning histories of failure with homework and self-organization, intolerance of frustrations, procrastination etc.);
- Individual psychotherapy and/or group therapy with the aim to improve behavioral, social and functional impairments; and
- Long-term follow-up to avoid early discontinuation and repeated disappointing experiences.

There has been little scientific research on systematic treatment approaches for comorbid patients suffering from BPD and ADHD, and more research in this area is needed.

## Conclusions

ADHD and BPD share some clinical features, particularly impulsivity and emotional instability. These disorders often co-occur. Patients with both diagnoses have more pronounced difficulties which are intertwined and often difficult to treat. In particular, impulsivity seems to be a severely impairing characteristic of patients suffering from both disorders. In BPD, impulsivity is primarily driven by affective and interpersonally sensitive aspects. In ADHD, deficits in attentional and cognitive processing account for behavior inhibition problems, referred to as impulsivity. Systematic treatment programs for patients with both ADHD and BPD are lacking, and there is a need for more research in this area. To stimulate research in the field of co-morbid ADHD and BPD we consider it important to further address the questions of gene-environment interactions, vulnerability to emotional neglect and abuse, and to broaden knowledge about temperament and the risk for traumatic experiences. Future research on impulsivity should include direct comparisons between ADHD, BPD and comorbid ADHD + BPD groups. The field of emotion regulation research in ADHD needs to be expanded to better understand interrelations between executive function deficits as well as (under-)stimulation and emotion regulation. The family interactions between parents and children with ADHD need to be considered from an emotion regulation perspective. Treatment options for comorbid patients suffering from ADHD and BPD should be developed and evaluated. Current knowledge emphasizes that therapeutic approaches need to take both disorders into consideration. Moreover, therapeutic planning needs to carefully consider symptom domains and selection of therapeutic steps and their succession.

## References

[1] Biederman J, Petty CR, Evans M, Small J, Faraone SV (2010). How persistent is ADHD? A controlled 10-year follow-up study of boys with ADHD. Psychiatry Res.

[2] Biederman J, Petty CR, Clarke A, Lomedico A, Faraone SV (2011). Predictors of persistent ADHD: an 11-year follow-up study. J Psychiatr Res.

[3] Faraone SV, Biederman J, Mick E (2006). The age-dependent decline of attention deficit hyperactivity disorder: a meta-analysis of follow-up studies. Psychol Med.

[4] Mannuzza S, Klein RG, Moulton JL (2003). Persistence of attention-deficit/hyperactivity disorder into adulthood: what have we learned from the prospective follow-up studies?. J Atten Disord.

[5] Mick E, Byrne D, Fried R, Monuteaux M, Faraone SV, Biederman J (2011). Predictors of ADHD persistence in girls at 5-year follow-up. J Atten Disord.

[6] Spencer TJ, Biederman J, Mick E (2007). Attention-deficit/hyperactivity disorder: diagnosis, lifespan, comorbidities, and neurobiology. J Pediatr Psychol.

[7] Davids E, Gastpar M (2005). Attention deficit hyperactivity disorder and borderline personality disorder. Prog Neuropsychopharmacol Biol Psychiatry.

[8] Wender PH, Reimherr FW, Wood DR (1981). Attention deficit disorder (‘Minimal Brain Dysfunction’) in adults: a replication study of diagnosis and drug treatment. Arch Gen Psychiatry.

[9] Barkley RA, Murphy KR (2010). Deficient emotional self-regulation in adults with attention-deficit/hyperactivity disorder (ADHD): the relative contributions of emotional impulsiveness and ADHD symptoms to adaptive impairments in major life activities. J ADHD Relat Disord.

[10] Corbisiero S, Stieglitz RD, Retz W, Rosler M (2013). Is emotional dysregulation part of the psychopathology of ADHD in adults?. Atten Defic Hyperact Disord.

[11] Surman CB, Biederman J, Spencer T, Yorks D, Miller CA, Petty CR, Faraone SV (2011). Deficient emotional self-regulation and adult attention deficit hyperactivity disorder: a family risk analysis. Am J Psychiatry.

[12] Lampe K, Konrad K, Kroener S, Fast K, Kunert HJ, Herpertz SC (2007). Neuropsychological and behavioural disinhibition in adult ADHD compared to borderline personality disorder. Psychol Med.

[13] Sebastian A, Jacob G, Lieb K, Tuscher O (2013). Impulsivity in borderline personality disorder: a matter of disturbed impulse control or a facet of emotional dysregulation?. Curr Psychiatry Rep.

[14] American Psychiatric Association (2013). Diagnostic and Statistical Manual of Mental Disorders.

[15] Dilling H, Mombour W, Schmidt MH, WHO (2005). International classification of psychiatric disorders (ICD-10 Chapter V (F)).

[16] Wender PH, Wolf LE, Wasserstein J (2001). Adults with ADHD: an overview. Ann N Y Acad Sci.

[17] Martel MM (2009). Research review: a new perspective on attention-deficit/hyperactivity disorder: emotion dysregulation and trait models. J Child Psychol Psychiatry.

[18] Retz W, Stieglitz RD, Corbisiero S, Retz-Junginger P, Rosler M (2012). Emotional dysregulation in adult ADHD: what is the empirical evidence?. Expert Rev Neurother.

[19] Sobanski E, Banaschewski T, Asherson P, Buitelaar J, Chen W, Franke B, Holtmann M, Krumm B, Sergeant J, Sonuga-Barke E, Stringaris A, Taylor E, Anney R, Ebstein RP, Gill M, Miranda A, Mulas F, Oades RD, Roeyers H, Rothenberger A, Steinhausen HC, Faraone SV (2010). Emotional lability in children and adolescents with attention deficit/hyperactivity disorder (ADHD): clinical correlates and familial prevalence. J Child Psychol Psychiatry.

[20] Philipsen A (2006). Differential diagnosis and comorbidity of attention-deficit/hyperactivity disorder (ADHD) and borderline personality disorder (BPD) in adults. Eur Arch Psychiatry Clin Neurosci.

[21] Philipsen A, Feige B, Hesslinger B, Scheel C, Ebert D, Matthies S, Limberger MF, Kleindienst N, Bohus M, Lieb K (2009). Borderline typical symptoms in adult patients with attention deficit/hyperactivity disorder. Atten Defic Hyperact Disord.

[22] Wolff S, Stiglmayr C, Bretz HJ, Lammers CH, Auckenthaler A (2007). Emotion identification and tension in female patients with borderline personality disorder. Br J Clin Psychol.

[23] Murray ME (1979). Minimal brain dysfunction and borderline personality adjustment. Am J Psychother.

[24] Rey JM, Morris-Yates A, Singh M, Andrews G, Stewart GW (1995). Continuities between psychiatric disorders in adolescents and personality disorders in young adults. Am J Psychiatry.

[25] Fischer M, Barkley RA, Smallish L, Fletcher K (2002). Young adult follow-up of hyperactive children: self-reported psychiatric disorders, comorbidity, and the role of childhood conduct problems and teen CD. J Abnorm Child Psychol.

[26] Rasmussen P, Gillberg C (2000). Natural outcome of ADHD with developmental coordination disorder at age 22 years: a controlled, longitudinal, community-based study. J Am Acad Child Adolesc Psychiatry.

[27] Miller CJ, Flory JD, Miller SR, Harty SC, Newcorn JH, Halperin JM (2008). Childhood attention-deficit/hyperactivity disorder and the emergence of personality disorders in adolescence: a prospective follow-up study. J Clin Psychiatry.

[28] Jacob CP, Romanos J, Dempfle A, Heine M, Windemuth-Kieselbach C, Kruse A, Reif A, Walitza S, Romanos M, Strobel A, Brocke B, Schäfer H, Schmidtke A, Böning J, Lesch KP (2007). Co-morbidity of adult attention-deficit/hyperactivity disorder with focus on personality traits and related disorders in a tertiary referral center. Eur Arch Psychiatry Clin Neurosci.

[29] Miller TW, Nigg JT, Faraone SV (2007). Axis I and II comorbidity in adults with ADHD. J Abnorm Psychol.

[30] Matthies S, Van Elst LT, Feige B, Fischer D, Scheel C, Krogmann E, Perlov E, Ebert D, Philipsen A (2011). Severity of childhood attention-deficit hyperactivity disorder-a risk factor for personality disorders in adult life?. J Pers Disord.

[31] Cumyn L, French L, Hechtman L (2009). Comorbidity in adults with attention-deficit hyperactivity disorder. Can J Psychiatry.

[32] Rosler M, Retz W, Retz-Junginger P, Hengesch G, Schneider M, Supprian T, Schwitzgebel P, Pinhard K, Dovi-Akue N, Wender P, Thome J (2004). Prevalence of attention deficit-/hyperactivity disorder (ADHD) and comorbid disorders in young male prison inmates. Eur Arch Psychiatry Clin Neurosci.

[33] Rosler M, Retz W, Yaqoobi K, Burg E, Retz-Junginger P (2009). Attention deficit/hyperactivity disorder in female offenders: prevalence, psychiatric comorbidity and psychosocial implications. Eur Arch Psychiatry Clin Neurosci.

[34] Westmoreland P, Gunter T, Loveless P, Allen J, Sieleni B, Black DW (2010). Attention deficit hyperactivity disorder in men and women newly committed to prison: clinical characteristics, psychiatric comorbidity, and quality of life. Int J Offender Ther Comp Criminol.

[35] Fossati A, Novella L, Donati D, Donini M, Maffei C (2002). History of childhood attention deficit/hyperactivity disorder symptoms and borderline personality disorder: a controlled study. Compr Psychiatry.

[36] Philipsen A, Limberger MF, Lieb K, Feige B, Kleindienst N, Ebner-Priemer U, Barth J, Schmahl C, Bohus M (2008). Attention-deficit hyperactivity disorder as a potentially aggravating factor in borderline personality disorder. Br J Psychiatry.

[37] Bernardi S, Faraone SV, Cortese S, Kerridge BT, Pallanti S, Wang S, Blanco C (2012). The lifetime impact of attention deficit hyperactivity disorder: results from the National Epidemiologic Survey on Alcohol and Related Conditions (NESARC). Psychol Med.

[38] Distel MA, Carlier A, Middeldorp CM, Derom CA, Lubke GH, Boomsma DI (2011). Borderline personality traits and adult attention-deficit hyperactivity disorder symptoms: a genetic analysis of comorbidity. Am J Med Genet B Neuropsychiatr Genet.

[39] Lehn H, Derks EM, Hudziak JJ, Heutink P, van Beijsterveldt TC, Boomsma DI (2007). Attention problems and attention-deficit/hyperactivity disorder in discordant and concordant monozygotic twins: evidence of environmental mediators. J Am Acad Child Adolesc Psychiatry.

[40] Schwarze CE, Mobascher A, Pallasch B, Hoppe G, Kurz M, Hellhammer DH, Lieb K (2013). Prenatal adversity: a risk factor in borderline personality disorder?. Psychol Med.

[41] Pagura J, Stein MB, Bolton JM, Cox BJ, Grant B, Sareen J (2010). Comorbidity of borderline personality disorder and posttraumatic stress disorder in the U.S. population. J Psychiatr Res.

[42] Ball JS, Links PS (2009). Borderline personality disorder and childhood trauma: evidence for a causal relationship. Curr Psychiatry Rep.

[43] Biederman J, Petty C, Spencer TJ, Woodworth KY, Bhide P, Zhu J, Faraone SV (2013). Is ADHD a risk for posttraumatic stress disorder (PTSD)? Results from a large longitudinal study of referred children with and without ADHD. World J Biol Psychiatry.

[44] Rucklidge JJ, Brown DL, Crawford S, Kaplan BJ (2006). Retrospective reports of childhood trauma in adults with ADHD. J Atten Disord.

[45] Johnston C, Mash EJ, Miller N, Ninowski JE (2012). Parenting in adults with attention-deficit/hyperactivity disorder (ADHD). Clin Psychol Rev.

[46] Burke JD, Stepp SD (2012). Adolescent disruptive behavior and borderline personality disorder symptoms in young adult men. J Abnorm Child Psychol.

[47] Stepp SD, Burke JD, Hipwell AE, Loeber R (2012). Trajectories of attention deficit hyperactivity disorder and oppositional defiant disorder symptoms as precursors of borderline personality disorder symptoms in adolescent girls. J Abnorm Child Psychol.

[48] van Dijk F, Lappenschaar M, Kan C, Verkes RJ, Buitelaar J (2011). Lifespan attention deficit/hyperactivity disorder and borderline personality disorder symptoms in female patients: a latent class approach. Psychiatry Res.

[49] van Dijk FE, Lappenschaar M, Kan CC, Verkes RJ, Buitelaar JK (2012). Symptomatic overlap between attention-deficit/hyperactivity disorder and borderline personality disorder in women: the role of temperament and character traits. Compr Psychiatry.

[50] Chamberlain SR, Sahakian BJ (2007). The neuropsychiatry of impulsivity. Curr Opin Psychiatry.

[51] Dickman SJ (1990). Functional and dysfunctional impulsivity: personality and cognitive correlates. J Pers Soc Psychol.

[52] Evenden JL (1999). Varieties of impulsivity. Psychopharmacol (Berl).

[53] Moeller FG, Barratt ES, Dougherty DM, Schmitz JM, Swann AC (2001). Psychiatric aspects of impulsivity. Am J Psychiatry.

[54] Barkley RA (1997). Behavioral inhibition, sustained attention, and executive functions: constructing a unifying theory of ADHD. Psychol Bull.

[55] Alderson RM, Rapport MD, Kofler MJ (2007). Attention-deficit/hyperactivity disorder and behavioral inhibition: a meta-analytic review of the stop-signal paradigm. J Abnorm Child Psychol.

[56] Nigg JT, Silk KR, Stavro G, Miller T (2005). Disinhibition and borderline personality disorder. Dev Psychopathol.

[57] Rentrop M, Backenstrass M, Jaentsch B, Kaiser S, Roth A, Unger J, Weisbrod M, Renneberg B (2008). Response inhibition in borderline personality disorder: performance in a Go/Nogo task. Psychopathology.

[58] de Bruijn ER, Grootens KP, Verkes RJ, Buchholz V, Hummelen JW, Hulstijn W (2006). Neural correlates of impulsive responding in borderline personality disorder: ERP evidence for reduced action monitoring. J Psychiatr Res.

[59] Swirsky-Sacchetti T, Gorton G, Samuel S, Sobel R, Genetta-Wadley A, Burleigh B (1993). Neuropsychological function in borderline personality disorder. J Clin Psychol.

[60] Domes G, Winter B, Schnell K, Vohs K, Fast K, Herpertz SC (2006). The influence of emotions on inhibitory functioning in borderline personality disorder. Psychol Med.

[61] Volker KA, Spitzer C, Limberg A, Grabe HJ, Freyberger HJ, Barnow S (2009). Executive dysfunctions in female patients with borderline personality disorder with regard to impulsiveness and depression. Psychother Psychosom Med Psychol.

[62] Jacob GA, Gutz L, Bader K, Lieb K, Tuscher O, Stahl C (2010). Impulsivity in borderline personality disorder: impairment in self-report measures, but not behavioral inhibition. Psychopathology.

[63] Ferrer M, Andion O, Matali J, Valero S, Navarro JA, Ramos-Quiroga JA, Torrubia R, Casas M (2010). Comorbid attention-deficit/hyperactivity disorder in borderline patients defines an impulsive subtype of borderline personality disorder. J Pers Disord.

[64] Speranza M, Revah-Levy A, Cortese S, Falissard B, Pham-Scottez A, Corcos M (2011). ADHD in adolescents with borderline personality disorder. BMC Psychiatry.

[65] Krause-Utz A, Sobanski E, Alm B, Valerius G, Kleindienst N, Bohus M, Schmahl C (2013). Impulsivity in relation to stress in patients with borderline personality disorder with and without co-occurring attention-deficit/hyperactivity disorder: an exploratory study. J Nerv Ment Dis.

[66] Sonuga-Barke EJS (2005). Causal models of attention-deficit/hyperactivity disorder: from common simple deficits to multiple developmental pathways. Biol Psychiatry.

[67] Carpenter RW, Trull TJ (2013). Components of emotion dysregulation in borderline personality disorder: a review. Curr Psychiatry Rep.

[68] Baer RA, Sauer SE (2011). Relationships between depressive rumination, anger rumination, and borderline personality features. Personal Disord.

[69] Rosenthal MZ, Cheavens JS, Lejuez CW, Lynch TR (2005). Thought suppression mediates the relationship between negative affect and borderline personality disorder symptoms. Behav Res Ther.

[70] Klonsky ED (2007). The functions of deliberate self-injury: a review of the evidence. Clin Psychol Rev.

[71] Silbersweig D, Clarkin JF, Goldstein M, Kernberg OF, Tuescher O, Levy KN, Brendel G, Pan H, Beutel M, Pavony MT, Epstein J, Lenzenweger MF, Thomas KM, Posner MI, Stern E (2007). Failure of frontolimbic inhibitory function in the context of negative emotion in borderline personality disorder. Am J Psychiatry.

[72] Yen S, Zlotnick C, Costello E (2002). Affect regulation in women with borderline personality disorder traits. J Nerv Ment Dis.

[73] Crowell SE, Beauchaine TP, Linehan MM (2009). A biosocial developmental model of borderline personality: elaborating and extending Linehan’s theory. Psychol Bull.

[74] Nica EI, Links PS (2009). Affective instability in borderline personality disorder: experience sampling findings. Curr Psychiatry Rep.

[75] Leyro TM, Zvolensky MJ, Bernstein A (2010). Distress tolerance and psychopathological symptoms and disorders: a review of the empirical literature among adults. Psychol Bull.

[76] Coifman KG, Berenson KR, Rafaeli E, Downey G (2012). From negative to positive and back again: polarized affective and relational experience in borderline personality disorder. J Abnorm Psychol.

[77] Carlson EA, Egeland B, Sroufe LA (2009). A prospective investigation of the development of borderline personality symptoms. Dev Psychopathol.

[78] Arntz A, Appels C, Sieswerda S (2000). Hypervigilance in borderline disorder: a test with the emotional Stroop paradigm. J Pers Disord.

[79] Sieswerda S, Arntz A, Mertens I, Vertommen S (2007). Hypervigilance in patients with borderline personality disorder: specificity, automaticity, and predictors. Behav Res Ther.

[80] Domes G, Schulze L, Herpertz SC (2009). Emotion recognition in borderline personality disorder-a review of the literature. J Pers Disord.

[81] Marx I, Domes G, Havenstein C, Berger C, Schulze L, Herpertz SC (2011). Enhanced emotional interference on working memory performance in adults with ADHD. World J Biol Psychiatry.

[82] Berger A, Kofman O, Livneh U, Henik A (2007). Multidisciplinary perspectives on attention and the development of self-regulation. Prog Neurobiol.

[83] Bresner T, Moussa W, Reschke K (2009). Emotionsregulation von Erwachsenen mit Aufmerksamkeitsdefizit-/Hyperaktivitätsstörung (ADHS).

[84] Desman C, Schneider A, Ziegler-Kirbach E, Petermann F, Mohr B, Hampel P (2006). Behavioural inhibition and emotion regulation among boys with ADHD during a go-/nogo-task. Prax Kinderpsychol Kinderpsychiatr.

[85] Walcott CM, Landau S (2004). The relation between disinhibition and emotion regulation in boys with attention deficit hyperactivity disorder. J Clin Child Adolesc Psychol.

[86] Sjöwall D, Roth L, Lindqvist S, Thorell LB (2013). Multiple deficits in ADHD: executive dysfunction, delay aversion, reaction time variability, and emotional deficits. J Child Psychol Psychiatry.

[87] Musser ED, Backs RW, Schmitt CF, Ablow JC, Measelle JR, Nigg JT (2011). Emotion regulation via the autonomic nervous system in children with attention-deficit/hyperactivity disorder (ADHD). J Abnorm Child Psychol.

[88] Rash JA, Aguirre-Camacho A (2012). Attention-deficit hyperactivity disorder and cardiac vagal control: a systematic review. Atten Defic Hyperact Disord.

[89] Ebert D, Krause J, Roth-Sackenheim C (2003). ADHD in adulthood–guidelines based on expert consensus with DGPPN support. Nervenarzt.

[90] **National Institutes of Health Consensus Development Conference Statement: diagnosis and treatment of attention-deficit/hyperactivity disorder (ADHD)** *J Am Acad Child Adolesc Psychiatry* 2000, **39:**182–193. 10.1097/00004583-200002000-00018 10673829

[91] Kooij SJ, Bejerot S, Blackwell A, Caci H, Casas-Brugue M, Carpentier PJ, Edvinsson D, Fayyad J, Foeken K, Fitzgerald M, Gaillac V, Ginsberg Y, Henry C, Krause J, Lensing MB, Manor I, Niederhofer H, Nunes-Filipe C, Ohlmeier MD, Oswald P, Pallanti S, Pehlivanidis A, Ramos-Quiroga JA, Rastam M, Ryffel-Rawak D, Stes S, Asherson P (2010). European consensus statement on diagnosis and treatment of adult ADHD: the European network adult ADHD. BMC Psychiatry.

[92] Koesters M, Becker T, Kilian R, Fegert JM, Weinmann S (2009). Limits of meta-analysis: methylphenidate in the treatment of adult attention-deficit hyperactivity disorder. J Psychopharmacol.

[93] Hooberman D, Stern TA (1984). Treatment of attention deficit and borderline personality disorders with psychostimulants: case report. J Clin Psychiatry.

[94] van Reekum R, Links PS (1994). N of 1 study: methylphenidate in a patient with borderline personality disorder and attention deficit hyperactivity disorder. Can J Psychiatry.

[95] Golubchik P, Sever J, Zalsman G, Weizman A (2008). Methylphenidate in the treatment of female adolescents with cooccurrence of attention deficit/hyperactivity disorder and borderline personality disorder: a preliminary open-label trial. Int Clin Psychopharmacol.

[96] Durell TM, Adler LA, Williams DW, Deldar A, McGough JJ, Glaser PE, Rubin RL, Pigott TA, Sarkis EH, Fox BK (2013). Atomoxetine treatment of attention-deficit/hyperactivity disorder in young adults with assessment of functional outcomes: a randomized, double-blind, placebo-controlled clinical trial. J Clin Psychopharmacol.

[97] Ghanizadeh A, Freeman RD, Berk M (2013). Efficacy and adverse effects of venlafaxine in children and adolescents with ADHD: a systematic review of non-controlled and controlled trials. Rev Recent Clin Trials.

[98] Maneeton N, Maneeton B, Srisurapanont M, Martin SD (2011). Bupropion for adults with attention-deficit hyperactivity disorder: meta-analysis of randomized, placebo-controlled trials. Psychiatry Clin Neurosci.

[99] Schuppert HM, Timmerman ME, Bloo J, van Gemert TG, Wiersema HM, Minderaa RB, Emmelkamp PM, Nauta MH (2012). Emotion regulation training for adolescents with borderline personality disorder traits: a randomized controlled trial. J Am Acad Child Adolesc Psychiatry.

[100] Carter GL, Willcox CH, Lewin TJ, Conrad AM, Bendit N (2010). Hunter DBT project: randomized controlled trial of dialectical behaviour therapy in women with borderline personality disorder. Aust N Z J Psychiatry.

[101] Linehan MM, Armstrong HE, Suarez A, Allmon D, Heard HL (1991). Cognitive-behavioral treatment of chronically parasuicidal borderline patients. Arch Gen Psychiatry.

[102] Linehan MM, Comtois KA, Murray AM, Brown MZ, Gallop RJ, Heard HL, Korslund KE, Tutek DA, Reynolds SK, Lindenboim N (2006). Two-year randomized controlled trial and follow-up of dialectical behavior therapy vs therapy by experts for suicidal behaviors and borderline personality disorder. Arch Gen Psychiatry.

[103] McMain SF, Links PS, Gnam WH, Guimond T, Cardish RJ, Korman L, Streiner DL (2009). A randomized trial of dialectical behavior therapy versus general psychiatric management for borderline personality disorder. Am J Psychiatry.

[104] van den Bosch LM, Koeter MW, Stijnen T, Verheul R, Van den BW (2005). Sustained efficacy of dialectical behaviour therapy for borderline personality disorder. Behav Res Ther.

[105] Stoffers JM, Vollm BA, Rucker G, Timmer A, Huband N, Lieb K (2012). Psychological therapies for people with borderline personality disorder. Cochrane Database Syst Rev.

[106] Knouse LE, Cooper-Vince C, Sprich S, Safren SA (2008). Recent developments in the psychosocial treatment of adult ADHD. Expert Rev Neurother.

[107] Knouse LE, Safren SA (2010). Current status of cognitive behavioral therapy for adult attention-deficit hyperactivity disorder. Psychiatr Clin North Am.

[108] Philipsen A (2012). Psychotherapy in adult attention deficit hyperactivity disorder: implications for treatment and research. Expert Rev Neurother.

[109] Hesslinger B, Tebartz VE, Nyberg E, Dykierek P, Richter H, Berner M, Ebert D (2002). Psychotherapy of attention deficit hyperactivity disorder in adults–a pilot study using a structured skills training program. Eur Arch Psychiatry Clin Neurosci.

[110] Hirvikoski T, Waaler E, Alfredsson J, Pihlgren C, Holmstrom A, Johnson A, Ruck J, Wiwe C, Bothen P, Nordstrom AL (2011). Reduced ADHD symptoms in adults with ADHD after structured skills training group: results from a randomized controlled trial. Behav Res Ther.

[111] Philipsen A, Richter H, Peters J, Alm B, Sobanski E, Colla M, Munzebrock M, Scheel C, Jacob C, Perlov E, Tebartz van Elst L, Hesslinger B (2007). Structured group psychotherapy in adults with attention deficit hyperactivity disorder: results of an open multicentre study. J Nerv Ment Dis.

[112] Philipsen A, Graf E, Tebartz VE, Jans T, Warnke A, Hesslinger B, Ebert D, Gerlach M, Matthies S, Colla M, Jacob C, Sobanski E, Alm B, Rösler M, Ihorst G, Gross-Lesch S, Gentschow L, Kis B, Huss M, Lieb K, Schlander M, Berger M (2010). Evaluation of the efficacy and effectiveness of a structured disorder tailored psychotherapy in ADHD in adults: study protocol of a randomized controlled multicentre trial. Atten Defic Hyperact Disord.

[113] Hesslinger B, Philipsen A, Richter H (2003). Psychotherapie der ADHS im Erwachsenenalter: Ein Arbeitsbuch.

